# Gene Editing to Tackle Facioscapulohumeral Muscular Dystrophy

**DOI:** 10.3389/fgeed.2022.937879

**Published:** 2022-07-15

**Authors:** Virginie Mariot, Julie Dumonceaux

**Affiliations:** NIHR Biomedical Research Centre, Great Ormond Street Institute of Child Health and Great Ormond Street Hospital NHS Trust, University College London, London, United Kingdom

**Keywords:** FSHD, gene editing, CRISPR, cas9, therapy, TALEN, DUX4, muscle

## Abstract

Facioscapulohumeral dystrophy (FSHD) is a skeletal muscle disease caused by the aberrant expression of the DUX4 gene in the muscle tissue. To date, different therapeutic approaches have been proposed, targeting DUX4 at the DNA, RNA or protein levels. The recent development of the clustered regularly interspaced short-palindromic repeat (CRISPR) based technology opened new avenues of research, and FSHD is no exception. For the first time, a cure for genetic muscular diseases can be considered. Here, we describe CRISPR-based strategies that are currently being investigated for FSHD. The different approaches include the epigenome editing targeting the DUX4 gene and its promoter, gene editing targeting the polyadenylation of DUX4 using TALEN, CRISPR/cas9 or adenine base editing and the CRISPR-Cas9 genome editing for SMCHD1. We also discuss challenges facing the development of these gene editing based therapeutics.

## Introduction

In the last decade, the gene therapy field was significantly modified by the development and discovery of genome editing tools that are the transcription activator-like effector nuclease (TALEN) and the clustered regularly interspaced palindromic repeat (CRISPR). The advent of these technologies opened up new possibilities of treatments. For example, CAR-T-cell immunotherapy, in which patient’s T cells are engineered *ex vivo* to recognize the patient’s cancer cells, produced remarkable responses in patients and CRISPR are now being used to induce the donated T cells to produce CARs (CAR T Cells, 2022). Another example is transthyretin amyloidosis, caused by progressive accumulation of misfolded transthyretin (TTR) protein in tissues, for which the systemic injection of lipid nanoparticle encapsulating messenger RNA for Cas9 protein and a single guide RNA targeting TTR, leading to DNA cleavage of the TTR to prevent the production of the misfolded TTR protein, is currently being evaluated in a phase 1 trial (ClinicalTrials.gov number, NCT04601051). The interim data published recently showed the first clinical evidence that CRISPR gene editing could be done effectively inside the body and without major safety problems (Gillmore et al., 2021; Therapeutics, 2022). According to the clinical trial website (www.clinicaltrial.gov), 32 studies involving the CRISPR/cas9 technology are ongoing in different treatment areas including blood disorders, cancers, chronic infections, eye disease or protein-folding disorders, but no muscular dystrophies. Most muscular dystrophies dramatically impact the patient’s life, which can even lead to premature death due to cardiac failure or respiratory dysfunction. To date, there is currently no cure for muscular dystrophies but a variety of treatments can help to slow disease progression and to manage the condition including corticosteroid medications to help maintain muscle strength, corrective surgery or treating heart complications. The lack of effective therapy may be explained by the fact that more than 40 genes have been described to be involved in muscular dystrophies (Kaplan and Hamroun, 2015) resulting in a wide range of abnormalities in proteins of the extracellular matrix and basement membrane, sarcolemma, nuclear membrane, sarcomere and with enzymatic function (Mercuri et al., 2019). Moreover, the muscle being the body’s largest organ, representing about 40% of the body mass, leads to significant delivery issues. Finally, the large size of the mutated genes such as dystrophin (14 kb mRNA) in Duchenne Muscular Dystrophy (DMD) or dysferlin (6.2 kb mRNA) in limb–girdle muscular dystrophy type 2B (LGMD2B) and Miyoshi myopathy, challenges classical gene replacement therapies. Genome engineering represents an alternative therapy for muscular disorders and several approaches have been developed for different muscular dystrophies including DMD, Myotonic dystrophy type 1 (DM1), Oculopharyngeal muscular dystrophy (OPMD), and a few types of LGMD [for review see (Nelson et al., 2017)]. This review highlights recent advances in genome engineering and their therapeutic potential for the treatment of Facioscapulohumeral muscular dystrophy (FSHD). Thereafter, we will discuss whether gene editing could be used as therapeutic strategy for FSHD.

## Genome Editing Tools

Gene editing started with the discovery of meganucleases in the 1990s, which are endodeoxyribonucleases that recognizes double-strand specific sequence of 12–40 bp. This large recognition site results in a very high specificity (a 18 bp sequence occurs only once in the human genome) but at the same time, is also the main limitation since it is difficult to develop new meganuclease variants that specifically target a new sequence [for review see (Gonzalez Castro et al., 2021)]. The first easily engineered nuclease was the zinc finger nuclease (ZNF). Zinc finger nuclease is composed of a non-specific endonuclease domain of the Fok1 restriction enzyme combined with zinc finger DNA-binding domain which recognize triplets of base pairs (Mandell and Barbas, 2006), the specificity of binding being increased by the use of ZNF attached together. However, the production of an efficient ZFN is often laborious and expensive and ZNF-associated toxicity has been described in several studies (Cathomen and Keith Joung, 2008). Two genome editing technologies have emerged during the last decade and have revolutionized the landscape of genome engineering: the TALE and CRISPR technologies. Today, the CRISPR/cas9 is the world’s most common gene editing tool because of its ease to use and design.

### TALE and TALEN Genome Editing Technologies

**FIGURE 1:**
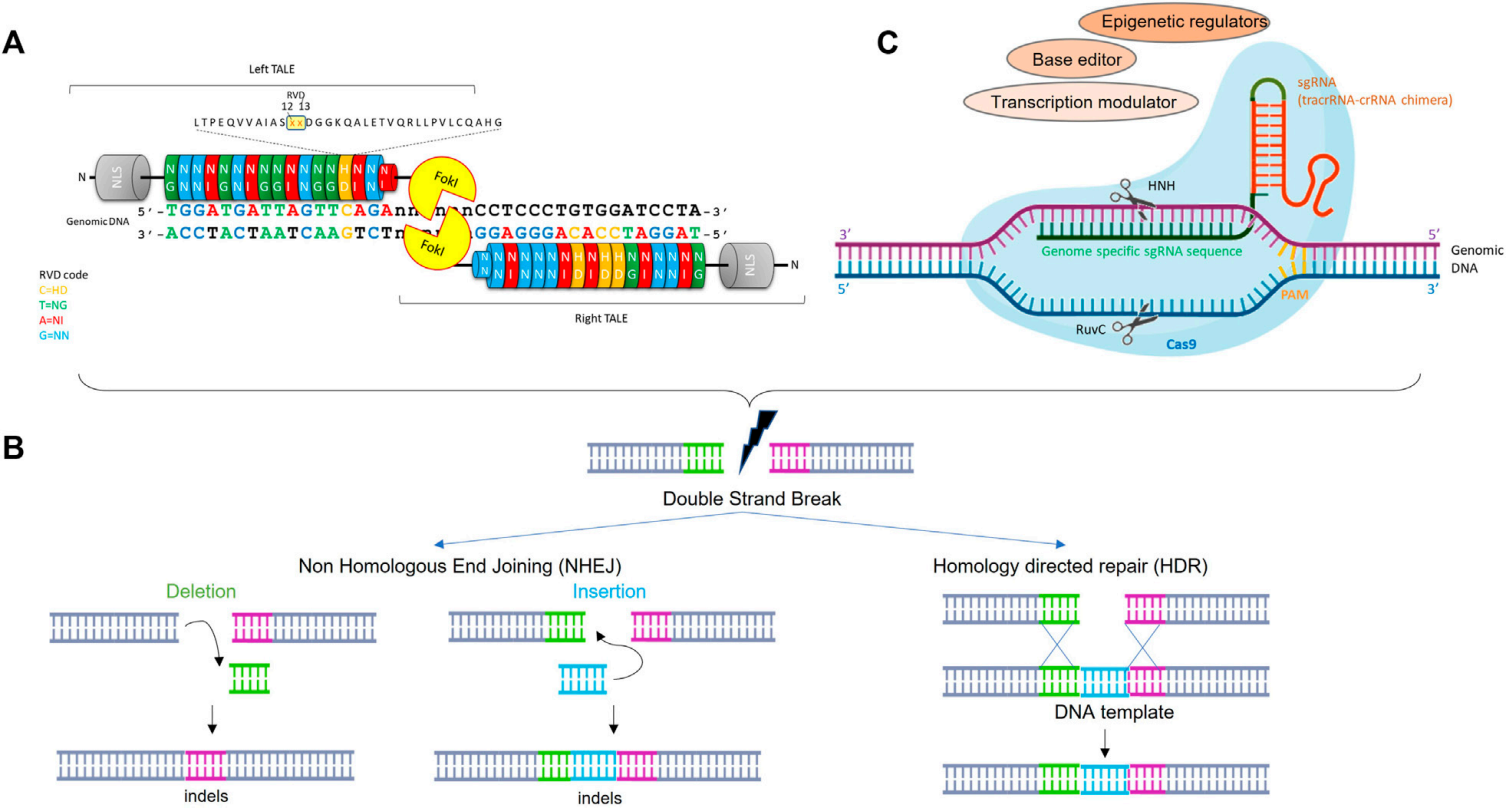
Schematic structure and activity of TALEN and CRISPR/cas9 for genome editing. **(A)**: TALENs and CRISPR/cas9 are engineered to bind a target sequence of interest by assembly from TALE repeat units specific to each base pair or simple cloning into sgRNA expression vector, respectively. **(B)**: Nuclease-induced genome editing. Double strand break can be repaired by Non-homologous end joining (NHEJ) or homology directed repair (HDR). NHEJ can lead to small insertions and deletions (indels) at the DBS site, whereas HDR can lead to more precise mutations from a DNA donor template. **(C)**: Schematic representation of the CRISPR-Cas9 system where HNH and RuvC represents 2 nuclease domains of Cas9. The single guide RNA (sgRNA) molecule directs Cas9 protein to a specific DNA target. The Cas 9 cleaves genomic DNA upstream to the PAM (Protospacer Adjacent Motif) sequence. The Cas9 can be fused to different modulators or editors to mediate gene expression regulation or base editing.

TALENs are a class of sequence-specific nucleases which results from fusion between a TAL effector transcription activator-like effector (TALE) DNA binding domain and the catalytic domain of the restriction endonuclease FokI (Mussolino and Cathomen, 2012) (Figure 1A). TAL DNA binding domains contain a highly conserved central region consisting of varying numbers of repeat units (ideally between 17 and 20) of 33–35 amino acids. Residues at position 12 and 13 in each repeat unit confer DNA binding site specificity, the most common pairs (HD, NG, NI and NN) accounting for each of the 4 nucleotides (C, T, A and G respectively) (Bogdanove and Voytas, 2011). Importantly, ordered assembly of the four basic repeats allows to easily construct a DNA binding domain specific to the sequence of interest and theoretically, any sequence in the genome can be targeted. Since FokI cleaves as a dimer, the TALENs function in pairs in which 2 monomers bind DNA sequences separated by a spacer that allows the formation of a Fok1 active dimer to cleave the genomic DNA and create a double-strand break (DSB) at a desired location that triggers genome editing (Cermak et al., 2011). Two major repair pathways have been described: the non-homologous end-joining (NHEJ), which typically results in small deletion or insertion called indels and the homology directed repair (HDR), which is a process of homologous recombination where a DNA template (such as single-strand oligonucleotide) is used to provide the homology necessary for precise repair of a DSB. Therefore, NHEJ is preferred for loss of function application (NHEJ-mediated repair efficacy is usually higher than HDR) (Figure 1B).

### The CRISPR-Cas9 Technology

The most recent gene editing and most widely used in academia laboratories is the CRISPR (clustered regularly interspaced short palindromic repeats)-Cas9 (CRISPR-associated protein 9) system. The CRIPSR-Cas9 was originally described as a putative RNA-interference-based immune system in prokaryotes (Makarova et al., 2006; Barrangou et al., 2007). After bacteriophage infection, short DNA sequences from the bacteriophage or fungi are incorporated into the host genome, within the CRIPSR locus. Upon bacteriophage reinfection, these sequences are transcribed into CRISPR RNAs (crRNA), which recruit a Cas protein to cleave the foreign DNA at sites complementary to the crRNA sequence (Sander and Joung, 2014). Foreign DNA recognition and cleavage can be achieved by 3 types of CRIPSR/Cas9 systems. Whereas types I and III involve a large multi-Cas protein complex that recognizes and cleaves DNA complementary to the crRNA, the type II system requires the presence of the Cas9 protein only.

Cas9 is a DNA endonuclease guided by 2 RNAs, the mature crRNA and the trans-activating crRNA (tracrRNA), which will form a crRNA/tracrRNA hybrid that guides Cas9 to cleave any DNA containing a sequence complementary to crRNA sequence that is adjacent to a short motif called protospacer adjacent motif (PAM) (Figure 1C) (Jinek et al., 2012). The demonstration of the capability of CRISPR-Cas9 to introduce targeted double-strand breaks in human cells associated with a simplified system, in which, crRNA and tracrRNA sequences are fused together into a single RNA chimera bearing the 20 nt sequence complementary to the target and the 42 nt stem loop structure required for Cas9 binding (Cho et al., 2013; Jinek et al., 2013; Mali et al., 2013), allowed the emergence of a revolutionary system used in biological research, human medicine (including cancer and genetic blood disorders), biotechnology, agriculture etc. By changing the complementary sequence to the target, this system can be used to target almost any sequence in the genome and, as for the TALEN, creates a DSB which will be repaired by HDR or NHEJ (Figure 1B).

Over the years, the CRISPR-Cas9 system has considerably evolved and repurposed and is no longer restricted to the cleavage of double strand DNA. Endonuclease deficient Cas9 proteins (dCas9) are now used to regulate the transcriptional activity of a target gene or edit it without double strand DNA break (Brezgin et al., 2019; Xu et al., 2020). The dCas9 was engineered to carry mutations in the 2 nuclease domains of Cas9 (NHN and RuvC), making it unable to cleave the DNA whereas it can still bind it. This dCas9 can be fused to different effector domains, including transcription activators or repressors, base editors or epigenetic regulators, leading to dCas9 derivatives with new functions (Figure 1C).

## Facioscapulohumeral Muscular Dystrophy

Facioscapulohumeral muscular dystrophy, also called Landouzy-Dejerine’ s disease from the names of the 2 French doctors who followed a family for 11 years and first described the principal clinical associated features, starts by a weakness and atrophy of selective groups of muscles including facial, shoulder and upper arm muscles. Most of the patients show symptoms in the teens. The disease progresses slowly and 20% of FSHD patients are wheelchair-bound (Tawil et al., 2014). Although FSHD is a skeletal muscle disease, extra muscular manifestations have been reported such as sensorineural hearing loss frequently due to cochlear dysfunction (Frezza et al., 2021) and retinal vasculopathy (Statland et al., 2013). These manifestations could be due to an altered expression of the protocaherin gene FAT1 (Caruso et al., 2013; Mariot et al., 2015). Respiratory function is usually not affected but patients with severe muscle weakness are at risk of deterioration (Teeselink et al., 2022).

**FIGURE 2:**
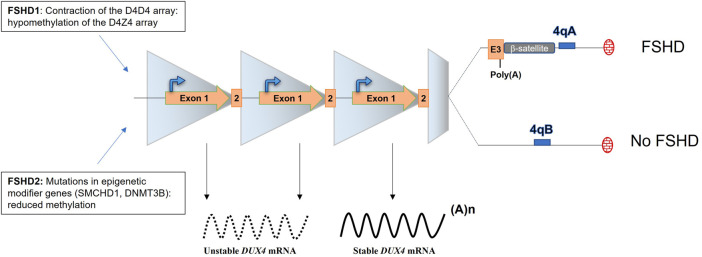
The locus D4Z4 and molecular mechanisms leading of FSHD. The D4Z4 locus is composed of 11–100 repeats in healthy individuals and is highly methylated. FSHD1 patients present a contraction of the D4Z4 array (1–10 units left) leading to chromatin relaxation. FSDH2 patients present also a chromatin relaxation that is mediated by mutations in epigenetic modifier genes. FSHD occurs when the D4Z4 array is relaxed and DUX4 expressed. DUX4 expression is uniquely associated with the 4qA haplotype, which carries the DUX4 p(A).

The actual consensus is that FSHD is caused by the aberrant expression of the DUX4 transcription factor in the skeletal muscle. DUX4 has been described to be expressed in the testis (Snider et al., 2009) and briefly during early embryonic development to play a role in the embryonic genome activation by driving different cleavage-specific gene expressions (De Iaco et al., 2017; Hendrickson et al., 2017) but is normally silenced in post mitotic tissues including muscles (Snider et al., 2009; Ferreboeuf et al., 2014a). In FSHD muscles, DUX4 expression is linked to the loss of repressive epigenetic marks and DNA hypomethylation of the D4Z4 repeats located on sub-telomeric part of the chromosome 4 (van Overveld et al., 2003; Zeng et al., 2009; Jones et al., 2015; Salsi et al., 2020) and to the presence of myogenic enhancers interacting with the DUX4 promoter (Himeda et al., 2014). Each D4Z4 unit carries the open reading frame of DUX4 (Gabriëls et al., 1999). In skeletal muscle, *DUX4* is composed of 3 exons and exons 1 and 2 are located in the D4Z4 unit whereas exon 3 is located outside of the D4Z4 array, in the sub telomeric part of the chromosome (Figure 2) (Sidlauskaite et al., 2020). Remarkably, 2 4qter variants (4qA and 4qB) of the sub-telomeric part of the chromosome exist in the general population and are equally distributed, but FSHD is uniquely associated with the 4qA allele which contains the polyadenylation signal (p(A), ATTAAA) of DUX4 (Lemmers et al., 2002). The importance of this DUX4 p(A) signal (PAS) in the disease onset was provided by the analysis of the sequence of chromosome 10 which shows 98.5% homology with chromosome 4 in its sub telomeric region. However, chromosome 10 is not associated with FSHD because of the presence of a single point mutation in the chromosome 10 DUX4 PAS sequence (ATCAAA) (Bakker et al., 1995; Lemmers et al., 2010).

Different mechanisms have been described to lead to the chromatin relaxation of the D4Z4 array. In the vast majority (95%) of FSHD patients (FSHD1, OMIM: 158,900), the de-repression of DUX4 is linked to the contracted array. FSHD1 patients carry between 1 and 10 repeats whereas in the normal population, this array usually varies from 11 to 100 (Figure 2) (Deutekom et al., 1993). Of note, at least 1 D4Z4 repeat is required to develop FSHD and an absence of the 4q telomeric region was observed in phenotypically normal cases (Tupler et al., 1996). In the remaining 5% of FSHD patients (FSHD2, OMIM: 158,901), mutations in epigenetic modifier genes including SMCHD1, DNMT3B or LRIF1, a known SMCHD1 protein interactor, have been reported (Figure 2) (Lemmers et al., 2012; van den Boogaard et al., 2016; Hamanaka et al., 2020; Jia et al., 2021). FSHD1 and FSHD2 are clinically undistinguishable (de Greef et al., 2010) and despite the fact that FSDH1 and 2 do not carry the same mutation, they both lead to chromatin relaxation and to the aberrant expression the DUX4 gene.

**FIGURE 3:**
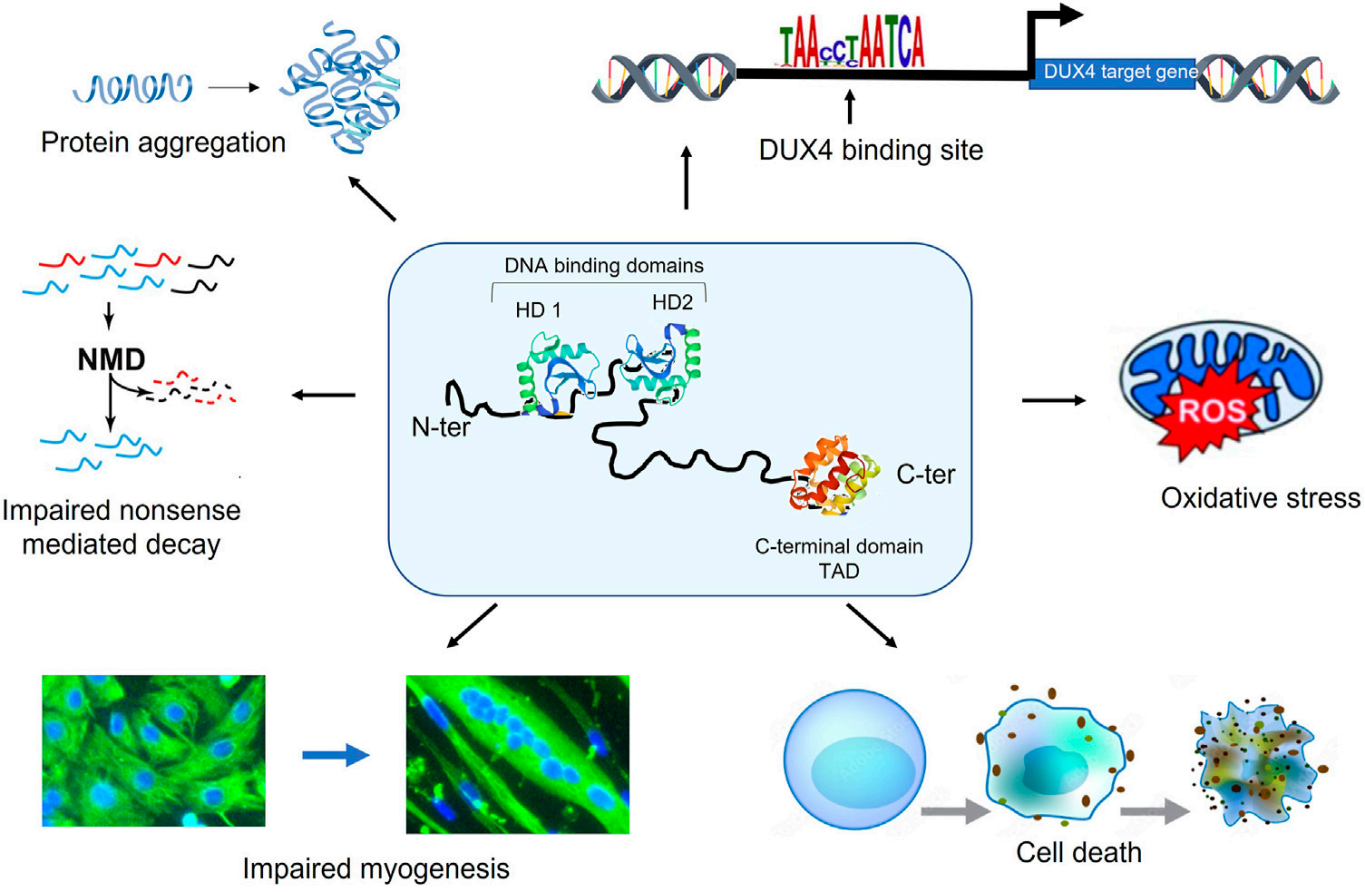
DUX4 is a toxic protein. DUX4 is composed of 2 DNA-binding homeodomains (HD1 and HD2) localized in its N-terminal region. The C-terminal part of the protein includes the transcription transactivation domain (TAD). DUX4 expression perturbs many cellular pathways involved leading to in myofiber death, increased sensitivity to oxidative stress, defects in myogenesis, muscle atrophy, inhibition of the non-sense mediated decay and the aberrant expression of hundreds of genes.

The pathologic role of DUX4 in FSHD onset and progression still needs to be deciphered. It is known that DUX4 is expressed as early as 14 weeks of development in FSHD foetuses (Broucqsault et al., 2013; Ferreboeuf et al., 2014a). Because the first symptoms usually appear during the teens, this suggests that FSHD could be at least partly attributed to the accumulation of DUX4 toxicity throughout life. DUX4 has been implicated in myofiber death, increased sensitivity to oxidative stress, defects in myogenesis, muscle atrophy, inhibition of the non-sense mediated decay, aberrant expression of hundreds of genes, etc (Figure 3). (DeSimone et al., 2017). Surprisingly, DUX4 mRNA is found in a very limited number of nuclei (about 1/1,000) but the DUX4 protein is present in up to 10% nuclei (Snider et al., 2010; Tassin et al., 2012; Block et al., 2013), which complicates its detection. How such a rare protein can trigger a muscular disease may be explained by the spreading of the DUX4 protein along the myofiber (Tassin et al., 2012; Ferreboeuf et al., 2014b).

## Therapeutic Strategies

To date, all the clinical trials for FSHD, with the exception of the Fulcrum Therapeutics trials ((NCT04003974, NCT04004000, NCT04264442), focused on non-specific approaches in order to reduce the oxidative stress, increase the muscle mass and strength or modulate the immune response to ameliorate the muscle homeostasis (Voermans et al., 2021). None of these non-targeted trials reach their primary endpoints and all were stopped. However, the last 10 years have made it possible to understand the causes of the disease, which allowed the development of multiple disease specific therapeutic approaches including strategies targeting DUX4 at DNA, mRNA, or protein levels, or downstream effects of DUX4. These different approaches have been reviewed elsewhere (Le Gall et al., 2020; Lim and Yokota, 2021) and in this review, we will focus on CRIPSR/Cas9-and TALEN- mediated genome editing and gene regulation approaches for FSHD. The CRISPR/Cas9 technology offers several advantages over the other approaches: one single injection is, in theory, sufficient to correct the genome permanently whereas oligonucleotide-based approaches or small molecules will have to be injected regularly throughout life. Moreover, since DUX4 expression is toxic and is associated with chromatin relaxation, Cas9 can be fused to different modulators or editors to inhibit the production of the DUX4 mRNA (Figure 1C). We will first review the different strategies already published and thereafter we will discuss the challenges shared by the different approaches. The different genome editing strategies for FSHD are summarized in Table 1.

**TABLE 1:** The gene editing therapeutic approaches for FSHD.

Cas9/TALEN	Strategy	Target	Study	Method	Gene expression (residual)	Reference
dCas9	KRAB	Prom/exon1 (*DUX4*)	*In vitro*	Lentivirus	50% (*TRIM43*)	Himeda et al (2016)
dSaCas9	SUV39H1	Prom/exon1 (*DUX4*)	*In vitro*	Lentivirus	50% (*TRIM43*)	Himeda et al (2021)
dSaCas9	HP1gamma	Prom/exon1 (*DUX4*)	*In vitro*	Lentivirus	50% (*TRIM43*)	Himeda et al (2021)
dSaCas9	MeCP2	Prom/exon1 (*DUX4*)	*In vitro*	Lentivirus	45% (*TRIM43*)	Himeda et al (2021)
dSaCas9	HP1α	Prom/exon1 (*DUX4*)	*In vitro*	Lentivirus	35% (*TRIM43*)	Himeda et al (2021)
dSaCas9	KRAB	Prom/exon1 (*DUX4*)	*In vivo*	AAV9 (IM)	50% (*Wfdc3*)	Himeda et al (2021)
SpCas9	HDR/KRAB	PAS (*DUX4*)	*In vitro*	Lentivirus	<20% (*DUX4*)	Das et al (2021)
SaCas9	ABE	PAS (*DUX4*)	*In vitro*	Transfection	No ABE activity	Sikrova et al. (2021)
CJCas9	ABE	PAS (*DUX4*)	*In vitro*	Transfection	No ABE activity	Sikrova et al (2021)
SpCas9	ABE	PAS (*DUX4*)	*In vitro*	Transfection	0%	Sikrova et al. (2021)
TALEN	HDR	PAS (*DUX4*)	*in vitro*	Transfection	15% (*TRIM43*)	Joubert et al. (2020)
SpCas9	DSB	PAS (*DUX4*)	*In vitro*	Transfection	Nd	Joubert et al. (2020)
NmCas9	D5B	PAS (*DUX4*)	*In vitro*	Transfection	Nd	Joubert et al. (2020)
SpCas9	DSB	*SMCHD1*	*In vitro*	Transfection	25% (*DUX4*)	Goossens et al (2019)

KRAB, Krϋppel-associated box; Prom, promoter; ABE, adenine base editor; DSB, double-strand break. PAS, polyadenylation signal; AAV, adeno associated virus; IM, intramuscular injection

Nd, not done

### Epigenome Editing Targeting DUX4 Gene and Promoter

The first study to demonstrate the potential of CRISPR/cas9 for the treatment of FSHD utilized a repurposed Cas9, namely the dead Cas9 (dCas9) (Table 2), fused to the Krüppel-associated box (KRAB, a potent transcriptional repressor that can be fused to heterologous DNA-binding protein repressor (Margolin et al., 1994)) to decrease DUX4 expression (Himeda et al., 2016). dCas9 variants carry mutations in their catalytic domains preserving their homing function but blocking the cleavage of genomic DNA (Qi et al., 2013). When co-expressed with a sgRNA, the complex dCas9/sgRNA can efficiently repress gene expression and repression efficiency can be increased, for example by improving the distance from the transcription start and the local chromatin state. The mechanism is based on a steric hindrance of target regions leading to an interference with RNA polymerase binding or elongation (Qi et al., 2013). However, if this model works well in prokaryotes, only a modest block of transcription is usually observed in mammalian cells probably because of the complexity of eucaryotic gene transcription that is driven by a variety of proteins involved in transcription regulation, such as activating and repressive transcription factors, the methylation status of the DNA, the presence of insulators etc. The fusion of the dCas9 to the KRAB domain, can robustly silence gene expression (Gilbert et al., 2013). In the article by Himeda and others, the authors utilized a dCas9-KRAB (from *Streptococcus pyogenes,* recognizing the NGG PAM motif) in the presence of single or multiple sgRNAs (Table 2). Primary FSHD cells were transduced with the lentiviral vectors expressing the dCas9-KRAB and individual sgRNAs targeting different regions of the D4Z4 repeat, as well as upstream or downstream sequences such as the exon 3 of DUX4. The authors observed that targeting the DUX4 promoter or exon 1 can reduce *DUX4* expression and the DUX4 downstream genes up to 45 and 60% of endogenous level respectively (Table 1). Another study also found a reduction of DUX4 and associated genes in the presence of dCas9-KRAB and sgRNA targeting the exon 3 of DUX4 (Das and Chadwick, 2021).

**TABLE 2:** Characteristics of the Cas9 used in the different therapeutic approaches for FSHD.

Abbreviation	Species	PAM motif	Packaging size	Strategy	Reference
SpCas9	*Streptococcus pyogenes*	5’NGG-3′	∼ 4.2 kb	Double strand break	Himeda et al (2021); Joubert et al (2020); Goossens et al (2019)
				KRAB	Himeda et al (2021); Das et al (2021)
				ABE	Sikrova et al. (2021)
SaCas9	*Staphylococcus aureus*	5’-NNGRRT-3’.	∼ 3.2 kb	ABE	Sikrova et al (2021)
dSaCas9	*Staphylococcus aureus*	5′-NNGRRT-3′	∼ 3.2 kb	Epigenetic regulator	Himeda et al (2016)
CjCas9	*Compylobocter jejuni*	5′-NNNVRYM-3′	∼ 2.9 kb	ABE	Sikrova et al (2021)
NmCas9	*Neisseria meningitidis*	5′-NNNNGATT-3′	∼ 3.5 kb	Double strand break	Joubert et al (2020)

Packaging site include Cas9 and sgRNA together.

N=A/G/C/T; R=G/A; V=G/C/A; Y=C/T; M=A/C.

ABE, adenine base editor.

This efficacy of the dCas9-KRAB system was also investigated *in vivo*. The dCas9 from *Staphylococcus aureus* (recognizing the NNGRRT PAM motif) was cloned into an AAV vector which, together with an AAV9-sgRNA targeting exon 1, was intramuscularly injected in the tibialis anterior of the ACTA1-MCM; FLExD bi-transgenic mice. After tamoxifen injection to induce *DUX4* transcription (Jones and Jones, 2018), expression of DUX4 was reduced up to 30% and transcript levels of 3 DUX4 downstream genes were also reduced (Himeda et al., 2021).

As expected, the repression of DUX4 transcription in the presence of the dCas9-KRAB was unlikely to be due to a steric hindrance of target regions, but instead mediated by the KRAB domain (Himeda et al., 2016). KRAB has been described to silence promoters by catalysing histone H3 lysine 9 methylation (H3K9me3), deacetylating histones and reducing RNA Pol II recruitment (Groner et al., 2010; Mlambo et al., 2018). KRAB recruits the KAP1 (KRAB-associated protein 1 (also known as TRIM28, Tif1β or KRIP-1) protein which in turn complexes with an array of epigenetic silencers including the heterochromatin protein 1 (HP1) and others (Friedman et al., 1996; Ryan et al., 1999; Abrink et al., 2001). In FSHD cells, the recruitment of dCas9-KRAB to the DUX4 promoter or exon 1 results in increased levels of KAP1, HP1α and HP1β and moderately decreased levels of activating H3K27ac mark and RNA Pol II recruitment (Himeda et al., 2016). As it was also shown that direct tethering of KAP1 to DNA was sufficient to repress transcription (Sripathy et al., 2006), when one of these epigenetic repressors are directly fused to the dCas9 and used to target the DUX4 promoter or exon 1, it also leads to a repression of DUX4 transcription in a similar manner (Table 1) (Himeda et al., 2021).

Epigenetic editing may also have some specific limitations. For example, KRAB/KAP1 recruitment has been described to induce a long-range repression through the spread of heterochromatin, tens of kilobases away from the KRAB bonding site (Groner et al., 2010). The combination of different epigenetic effector domains could also ameliorate the epigenetic silencing (O'Geen et al., 2017).

### Genome Editing Targeting the DUX4 PAS

The major role of the DUX4 PAS in the FSHD has been demonstrated in multiple articles (Bakker et al., 1995; Lemmers et al., 2010; Lemmers et al., 2022) and strategies aiming at destabilizing the *DUX4* mRNA by targeting its PAS using antisense oligonucleotides or U7snRNA have been successfully developed *in vitro* and *in vivo* (Chen et al., 2016; Marsollier et al., 2016; Ansseau et al., 2017; Lu-Nguyen et al., 2021; Rashnonejad et al., 2021; Lu-Nguyen et al., 2022). Targeting DUX4 polyadenylation signal is of interest because it is in exon 3, which is present in all the pathological *DUX4* mRNA isoforms in the muscle tissue, but absent in the isoforms expressed in the testis or during the early embryonic development (Snider et al., 2010; Sidlauskaite et al., 2020). The poly(A) site is thus a prime target for a TALEn or CRISPR application. Both CRISPR/cas9-and TALEn-based strategies have been developed to either inserting double strand breaks to delete this region (Das and Chadwick, 2021) or insert the target sequence of miR-1 into the DUX4 PAS (Joubert et al., 2020) (since miR-1 is massively upregulated in myotubes (Guller and Russell, 2010), which may be sufficient to inhibit translation of any *DUX4* residual mRNA), or triggering an adenine to guanine substitution with the PAS sequence (Sikrova et al., 2021). The difference in these approaches it that the first one relies on the DSB whereas the last one creates a single-strand break only.

**FIGURE 4:**
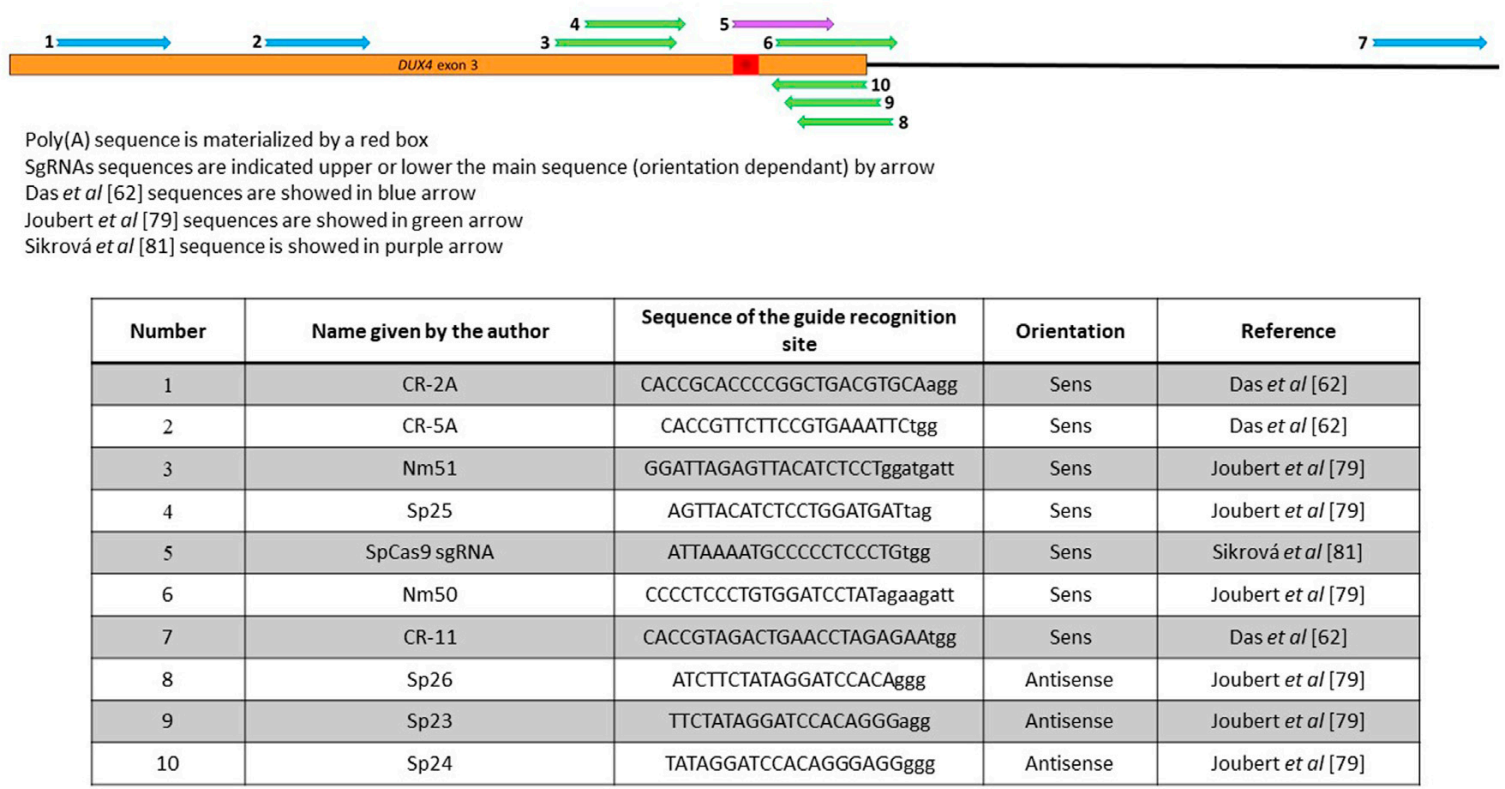
Position of the different sgRNAs used that target the 3′UTR of DUX4. The DUX4 poly(A) sequence is materialized by a red box. SgRNAs sequences are indicated upper or lower the main sequence (orientation dependant) by arrow. Sequences in Das 2021, Joubert 2020 or Sikrová 2021 are indicated in blue, green, purple respectively. Sequences are indicated in the lower part of the figure.

In the DSB-based strategies, immortalized FSHD myoblasts were either stably transduced with a combination of 1 lentiviral vector coding spCas9 and 2 lentiviral vectors coding a combination of sgRNAs upstream and downstream the poly-A site (Das and Chadwick, 2021) or transiently transfected with 2 plasmids coding the different TALEN pairs in the presence of the oligonucleotide (Figure 4) (Joubert et al., 2020). In the first case, the idea was to introduce a large deletion, which will be repaired by NHEJ. Deletions of 230–300 nt including the DUX4 PAS were reported, leading to a downregulation of *DUX4* and ZSCAN4 and TRIM43 DUX4 downstream genes (Table 1). It was not possible to assess the efficacy of this genome editing because the cells were analysed in bulk after transduction by the lentiviral vectors.

When myoblasts were transiently transfected with the TALEN pairs recognizing sequences surrounding the DUX4 PAS (Figure 4), the aim was to favour homology directed repairs (Joubert et al., 2020). The insertion of the oligonucleotide was observed, but with a very low efficiency (only few clones carrying an edited gene were isolated), which may be explained by the fact that the cells were not transduced or selected using antibiotics. These insertions lead to a decrease of the genes downstream of DUX4 (Table 1). However, HDR has been reported to be very inefficient in post-mitotic tissues such as skeletal muscle (Heyer et al., 2010; Lin et al., 2014), so this strategy is not expected to be very efficient if applied *in vivo* to muscle tissue.

Interestingly, 3′RACE nested PCR using forward primers located in exon 3 showed a redirection of the cleavage site in the modified clone, ∼40 nt upstream of the regular DUX4 PAS (Joubert et al., 2020). Alternative polyadenylation is a well described process and in mammals, a very large majority of transcripts have at least 2 alternative PAS, leading to different cleavage site and mRNA (Marsollier et al., 2018). These alternative PAS are driven by the presence of PAS motifs, but no alternative known PAS sequence was identified in the DUX4 upstream sequence. Remarkably, this alternative PAS was already described by the same group when an oligonucleotide directed against the DUX4 PAS and cleavage site was used to downregulate DUX4 (Marsollier et al., 2016).

One group reported an adenine base editing of the DUX4 PAS. The adenine base editing system consists of a SpCas9 nickase fused to an adenine base editor (ABE) that performs an A to G editing in the presence of a sgRNA (Gaudelli et al., 2017). Nickases are usually used to increase the specificity of cleavage. They carry a mutation in one of the 2 Cas9 nuclease domains and therefore still can bind the DNA but instead of cutting both DNA strand, can create a single break only. Consequently, 2 nicking Cas9s must be used to effectively create a double strand cleavage, which on one hand greatly lowers off target effects and allows a precise genome but on the other hand complicates the delivery because the 2 Cas9s are required in the same nucleus [for review see (Trevino and Zhang, 2014)]. In the article by Sikrová et al., SpCas9 nickase carries 1 mutation, D10A, in the RuvC cleavage domain of the Cas9, and was fused to either the ABE7.10 or the ABEmax version of the adenine base editor (ABE) (Table 2), which differs by modification of nuclear localization signals and codon usage thus improving its nuclear localization and expression (Koblan et al., 2018; Rees and Liu, 2018). This approach presents the advantage not to depend on the creation of double-strand breaks in DNA and therefore does not rely on the HDR pathway. As base editing is associated with excision repair or mismatch repair, it can occur in most cell-cycle phase and therefore can be performed in both dividing and non-dividing cells. The aim was to permanently modify the DUX4 PAS (ATTAAA) by editing one of the last 3 adenines into guanine to inactivate it using specific primers (Figure 4). Multiple clones (18% edited clones) carrying the desired mutations (A to G editing but also small deletions) were obtained using FSHD1 or FSHD2 cell lines and levels of *DUX4* and its target genes were dramatically reduced but not completely abolished (Table 1). Interestingly, the authors identified that 2 different cleavage sites (CS) in the edited clones, located before (proximal) or after (distal) the canonical CS. Remarkably, the proximal CS is the one previously described by Marsollier et al. (2016) and observed after editing the PAS with TALEN pairs (Joubert et al., 2020). The distal CS was only found in edited FSHD1 clones. This residual DUX4 mRNA is expressed at lower levels but no one knows if this level is sufficient to still drive muscle atrophy and muscle fibre death. One of the most crucial questions that the next clinical trials will face is probably: how many myofibers will need to be corrected, and how much of a reduction in DUX4 expression will be required to provide a functional benefit?

One of the limitations of adenine base editing approaches is the size of the ABE-spCas9 insert. SpCas9 is 4.2 kb long and ABE7.1 is 1.2Kb, which together exceed the limited capacity of AAV vectors (4.7 kb). AAV vectors being the leading strategy to develop gene therapy product to muscle, the use of smaller Cas9 orthologs may overcome this limitation. Orthologs such as SaCas9 (from *Staphylococcus aureus*) (Ran et al., 2015) or CjCas9 (from *Campylobacter* jejuni) (Kim et al., 2017) with gene size of 3.2kb and 2.95 Kb respectively may be used to overcome this challenge. However, when the Cas9 orthologs SaCas9 or CjCas9 were fused to ABEmax to target the DUX4 p(A), no adenine base editing activity was noted (Table 1) (Sikrova et al., 2021). This could be due to a more complex PAM requirement making it more difficult to find suitable target sequence.

### CRISPR-Cas9 Genome Editing for SMCHD1

The CRISPR-Cas9 genome editing was used to repair a pathogenic intronic SMCHD1 variant in patient myoblasts (Goossens et al., 2019). Mutations in SMCHD1 gene are associated with FSHD and SMCHD1 contributes to the DUX4 repression by directly binding to the D4Z4 array (Lemmers et al., 2012). The authors focused on a SMCHD1 variant leading to the exonisation of 53 bp (due to a deep intronic variant at position c.4347–236A > G) and to aberrant transcripts carrying a pseudo exon between exon 34 and 35 leading to a premature stop codon in exon 35. This mutation is likely to cause SMCHD1 haploinsufficiency. Genome editing was mediated by lentiviral transduction of spCas9 and guide RNAs into primary myoblasts (Table 2). A 407 bp genomic deletion in intron 34, which includes the deep intronic variant, was observed, leading to a higher expression of SMCHD1 and reduced expression of DUX4. Interestingly, no increased CpG methylation at DR1 was observed in the edited clones, which is consistent with the observation that SMCHD1 is involved in *de novo* methylation of the D4Z4 array but not in DNA methylation maintenance (Dion et al., 2019). The data presented in this article suggest that increasing level of wild-type SMCHD1 in patients presenting a SMCHD1 haploinsufficiency may result in DUX4 repression.

## Challenges

### Challenges Specific to FSHD

- The D4Z4 array. The genome editing technologies show great promise for human health in general and genetic diseases in particular. FSHD is no exception. The different approaches presented in this review have shown that several strategies are conceivable. Of note, none of them propose to remove the entire D4Z4 region. Such as strategy might not be suitable since numerous D4Z4 homologs are in different sites in the human genome (Chromosomes 3, 13, 14, 15, 21, 22 and Y) and the DUX4 gene exists in hundreds of copies (Himeda et al., 2015). In this case, targeting the D4Z4 repeat might lead to multiple DNA breaks, which may be associated with genomic instability and off-target effects.
- FSHD1 and FSDH2. The different strategies presented in this review target the 2 most important FSHD-causing genes, namely DUX4 and SMCHD1. The aberrant expression of DUX4 is observed in both FSHD1 and 2 patient biopsies and cell culture whereas mutations in SMCHD1 are mainly observed in FSHD2 patients. Few FSHD1 patients present both a D4Z4 contracted array and mutations in SMCHD1, but in this case, these mutations act as a modifier of disease severity only (Sacconi et al., 2013) and it is unknown if increased levels of wild type SMCHD1 will decrease DUX4 expression. Moreover, as more than 180 FSHD associated SMCHD1 variants have been described so far (Lemmers et al., 2019), a strategy based on CRISPR-Cas9 genome editing of a mutated SMCHD1 gene seems difficult to envisage in a near future. For all these reasons, targeting DUX4 seems to be the best option.
- The DUX4 gene. The 3 exons of the DUX4 gene were targeted, as well as the promoter and the p(A). Targeting the promoter or transcriptional start site using a dCas9 fused to a repressor has the advantage of physically impeding the transcription process without altering the DNA sequence itself but on the other hand, the dCas9-KRAB systems also may allow only temporary gene silencing, particularly in proliferating tissues and dividing cells. One may think that it might not be a major challenge for FSHD because skeletal muscle is a post mitotic tissue, but a long term follow up after dCas9-KRAB administration needs to be performed.
- DUX4 expression. Even if many of the articles searching for a therapeutic approach for FSHD targeting DUX4 look at DUX4 and DUX4 network gene levels as an outcome measure, no one knows how much reduction will provide a clinical benefit or slow the disease progression. Since DUX4 is expressed at very low levels in the muscle biopsies, it is expected that a small decrease should be beneficial.
- Satellite cell editing. FSHD patients present an atrophy of their muscles and regenerative myofibers are found in the majority of muscle biopsies from quadriceps or tibialis anterior (Banerji et al., 2020). In healthy individuals, muscle fibres are regenerated after injury, but in FSHD patients, it was shown that 76 and 91% of muscle biopsies from quadriceps and tibialis anterior respectively show regenerative myofibres. As the disease progress slowly, satellite cells may be little stimulated. However, life-long maintenance and repair of muscle tissue is mediated by satellite cells which undergo division after activation, give rise to myogenic progenitors that first proliferate, and eventually differentiate through fusion to damaged fibres to reconstitute fibre integrity, which could alter DUX4 silencing. An editing of satellite cells was reported in a Duchenne muscular dystrophy mouse model after postnatal injection of an AAV9 vector (Long et al., 2016).
- Absence of large animal model: different animal models have been published including several mouse, *drosophila* and zebrafish models (Le Gall et al., 2020) but no large model. Large models share similar pathogenic mechanisms with human patients because they are more analogous to humans in regard to body size, organ size, and lifespan. They are also less inbred when compared to rodents. Moreover, United States Federal Food and Drug Administration (FDA) and the European Medicines Agency (EMA), recommend the use of large animal models to evaluate efficacy, durability, dose response, degradation and safety of advanced therapeutic medicinal products (ATMPs), (Ribitsch et al., 2020). Large animal models are already successfully used for other muscular dystrophies including DMD, myotubular myopathy or SMA (Barthelemy et al., 2014; Duque et al., 2015; Barthelemy et al., 2019)

### Challenges not Specific to FSHD

- Delivery: AAV is the vector of choice to target skeletal muscle. They present multiple advantages: they are not associated with any human disease, they can be easily engineered for specific applications, they efficiently transduce the muscle tissue and show a long term transduction of non-dividing cells, and 3 AAV-based medicinal products have been already approved by the FDA, including one for the treatment of a neuromuscular disorder, Zolgensma (Onasemnogene abeparvovec-xioi) for spinal muscle atrophy (Hoy, 2019). AAVs also show drawbacks: they present a limited cloning capacity (4.7 kb). The SaCas9, which is compact (∼3.2 kb), became the preferred Cas9 variant for *in vivo* application. SpyCas9, one of the widely used Cas9, is 4.2 kb long and therefore can be cloned into an AAV but this leaves 0.5 kb only for regulatory elements including the promoter and p(A) and other genome editing components must be cloned into a separate vector. The consequence of a dual-vector approach is that genome editing can be achieved only if the 2 AAVs transduce the same cell. When Cas9 are fused with effectors such as ABE, the size of the insert to be cloned is 5.3 Kb, thus making it impossible to clone in one single vector. Strategies aiming at splitting a large transgene into 2 smaller segments that can be cloned into individual AAV vector have recently emerged and include the overlapping, trans-splicing, hybrid or intein approaches (Patel et al., 2019; Wang et al., 2022). These strategies have been already applied on animal models for muscular dystrophies such as limb–girdle muscular dystrophy type 2B and Miyoshi myopathy or DMD (Lostal et al., 2010; Kodippili et al., 2018). A split-intein ABE AAV approach has already been recently described after systemic injection of 2 × 10^12^vg (viral genomes) and an A to G editing of 9% was observed in the skeletal muscle (Levy et al., 2020). Another possibility is to design minimized regulatory cassette allowing the cloning of the Cas9 fused to repressors or modulators into one single AAV (Himeda et al., 2021).

Non-viral delivery vectors are also in development for therapeutic genome editing. Liposomes, polymers, extracellular vesicles and cell-penetrating peptides are at either preclinical or clinical stages and they can interact with cargo to form nanoparticles (Xu et al., 2019). They show the advantages of having the capability to deliver all the CRISPR components within 1 unique vector, to allow repeated injections and they have a low immunogenicity. However, they also present several challenges, among them the delivery into a specific cell type (which can be modulated by the addition of various moieties such as aptamer or Ab), the efficient intracellular release of the Cas9 into the cytosol after the receptor-mediated endocytosis, and the efficient nuclear entry (Behr et al., 2021).

- Immune response: Both the AAV vector and the Cas9 present some limitations due to pre-existing immunities. Pre-existing immunities to AAV are often found in humans, excluding a large proportion of patients from enrolment (Boutin et al., 2010). The innate and adaptative immune response directed against the vector restricts its therapeutic use [for review see (Buscara et al., 2020; Ronzitti et al., 2020; Shirley et al., 2020)] and several strategies are under development, such as the capsid specific removal of circulating Ab using plasmapheresis (Bertin et al., 2020) or the use of the use of an IgG-cleaving endopeptidase from *Streptococcus* pyogenes (IdeS) to cleave human IgG to decrease pre-existing AAV Ab (Leborgne et al., 2020). Most of the clinical trials include the presence of AAV binding antibodies in serum as exclusion criteria. This could lead to the exclusion of many FSHD patients, because FSHD patients would be treated during adulthood while SMA type1/2 or DMD patients, who are treated during childhood, are less impacted by the presence of anti AAV antibodies. Pre-existing immunity to Cas9 has been also reported as 78% of humans exhibit an immune response toward SaCas910 and 58%–96% toward SpCas9 (Charlesworth et al., 2019; Wagner et al., 2019), thus raising important safety and efficacy concerns as pre-existing immunity could lead to the elimination of the transduced cells and to the induction of a cytotoxic CD8^+^ T ell response to Cas9 expressing cells (Li et al., 2020) Cas9-specific immune responses was recently investigated in canine models of DMD following intramuscular and intravenous AAV-CRISPR therapy. Whereas dystrophin expression was observed at 3 weeks post injection, it was substantially reduced at 6 weeks with the presence of T-cell infiltration, muscle cytokine elevation, and muscle cell death with active killing detected by the presence of granzyme B + T-cells. An elevation of the serum Cas9 was also reported (Hakim et al., 2021). Importantly, this Cas9 immunity was not bypassed by strategies including tissue specific promoter or prednisolone immune suppression, which are commonly used.
- Off-targets: Although extremely powerful, the CRISPR/cas9 system shows several major limitations for *in vivo* applications. The off-target effect is one of the major concerns, as studies have revealed that Cas9 binds to unintended genomic sites (Teeselink et al., 2022). *In silico*, *in vitro* and *in vivo* techniques have been developed to detect off-target effects of Cas9. Diverse methods for reducing off-targets have been proposed including (1) hyper accurate variants of Cas9 (Snider et al., 2009; De Iaco et al., 2017), (2) the incorporation of chemical modifications in the guide RNA, the optimization of guide designs such as the truncation or extension at the 5′ends of gRNA, or the GC content (Ferreboeuf et al., 2014a), (3) the modification of the Cas9 system by limiting the expression of the Cas9 or gRNA or the use of mutated Cas9 nickases, and (4) to change the delivery system to specifically target the cell type of interest or restrict the transgene expression using cell specific promoters [for review see (Zhang et al., 2021)]. On target editing has been also described including chromosome rearrangements, AAV integration, or large deletion around the target site that may be profiled to assess genome editing outcome and safety.
- PAM sequence: the requirement for a PAM sequence next to the target sequence may prevent the targeting of specific sequences with the precision that is necessary for various genome-editing applications. In one of the most commonly used Cas9, the *Streptococcus pyogenes* (SpCas9), the PAM sequence is a 3 nt motif 5′-NGG-3′ where N is any nucleotide. Other nucleases, such as the Cas9 from *Staphylococcus aureus* (SaCas9) or *Campylobacter* jejuni (CjCas9) recognize more complex PAM motifs (NNGRRT and NNNVRYM respectively), restricting the ability to target any sequence with the CRISPR technology. Efforts were made to alter PAM recognition to increase the range of genome editing (Kleinstiver et al., 2015a; Kleinstiver et al., 2015b; Hu et al., 2018). Alternatively, the field is also searching for PAM-free nucleases or for a repertoire of nucleases that collectively recognize every possible sequences (Uddin et al., 2020; Collias and Beisel, 2021).

## Conclusions and Perspectives

The CRISPR/cas9 technology gives great hope for monogenic diseases, and FSHD is no exception. For the first time, this technology allows the prospect of a cure for FSHD by permanently altering genomic DNA in cells. The rapidity which the field has evolved is impressive and new application fields have emerged. New base editors have been developed such as the ABE8e which shows greatly increased editing efficiency relative to that of ABE7.10 (Richter et al., 2020). More flexible CRISPR/Cas technology with no PAM requirements is emerging (Collias and Beisel, 2021). Another example concerns the RNA knockdown application of the CRISPR, which has recently emerged by using the Cas13b ortholog from Prevotella sp. P5-125 (PspCas13b) (Cox et al., 2017). The Cas13 functions similarly to Cas9, using a gRNA, but targets RNA instead of DNA. RNA editing has advantages over DNA editing: it does not require homology directed repair (HDR) machinery and could thus be used in skeletal muscles. Moreover, Cas13 does not require a PAM sequence next to the target sequence and it cannot edit the genome and does not induce genomic off-targets. Therefore, this system holds the promise to complement or replace RNA interference approaches, but it is essential to determine if it offers a better specificity and efficiency compared to RNAi. The CRISPR/Cas13 tool requires a dual-AAV vector whereas a shRNA system requires only 1 vector. The major advantage of the Cas13 technology is that it does not rely on the DICER pathway and therefore can be used to target lncRNAs (Zhang et al., 2020). Prime editors, which are generated by fusion of Cas9 nickase with a modified reverse transcriptase to perform high efficiency of editing by small insertions or deletions could also be an option to decrease DUX4 levels (Scholefield and Harrison, 2021).

FSHD is a prime target to apply the CRISPR/Cas technology. Different CRIPSR-mediated technologies can be applied to FSHD, which do not necessary require a specific nucleotide change. Results are encouraging bur many challenges remain such as how to bypass the anti Cas9 immune response or how to assess the presence of genomic off-targets? Epigenome editing, because it does not edit the genome, could be part of the answer but needs to be tested in large animal before moving to humans. Many FSHD-related questions also remain; among them, how much DUX4 reduction will provide a clinical benefit and how long the effects observed after a CRIPSR strategy will last in humans. If the number of CRISPR clinical trials is growing, most of them are still in early stages and long-term safety consequences are not yet known.
